# Surgical repair for left ventricular apical aneurysm without coronary artery disease

**DOI:** 10.1016/j.xjtc.2025.06.019

**Published:** 2025-06-28

**Authors:** Satoshi Numata, Tomohito Nakashima, Tatsuro Gondai, Takuma Kobayashi

**Affiliations:** Department of Cardiovascular Surgery, Kyoto Prefectural University of Medicine, Kyoto, Japan

**Figure:**
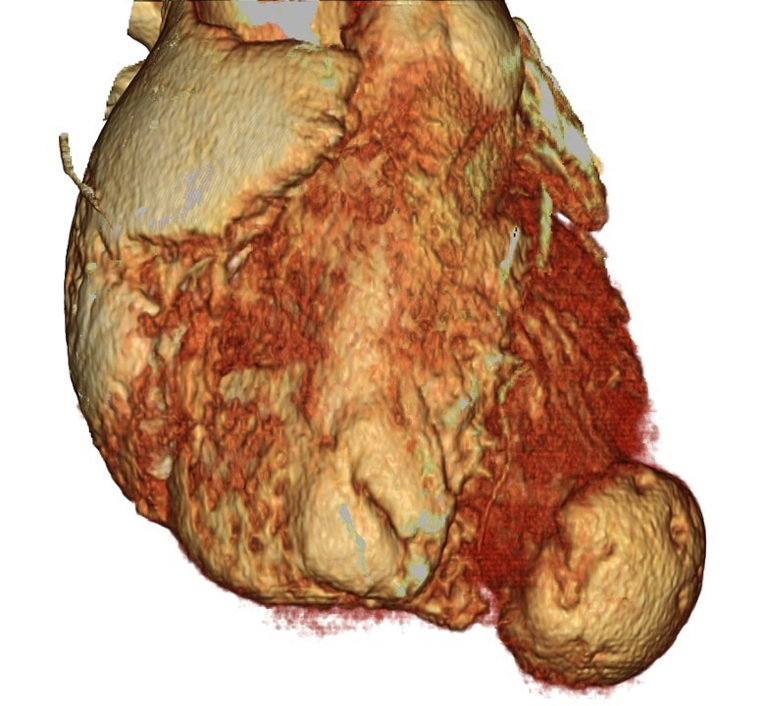
Left ventricular apical aneurysm without coronary artery disease.

**Central Message**

For repairing left ventricular aneurysm without coronary artery disease, left ventricular restoration procedure ELIET (endocardial linear infarct exclusion technique) is one useful option.

The most common cause of left ventricular aneurysm is coronary artery disease, but it also can occur due to trauma, sarcoidosis, and other factors. Here we report a case of surgical treatment of a left ventricular aneurysm located at the apex without significant coronary artery stenosis.

## Case Report

**Figure 1:**
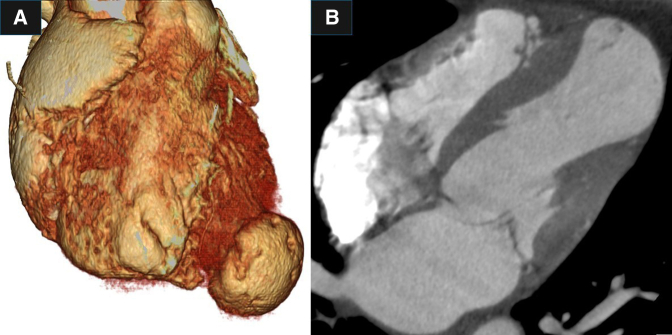
A, Three-dimensional reconstruction of preoperative computed tomography scan. B, Thin-slice image of preoperative computed tomography scan.

**Figure 2:**
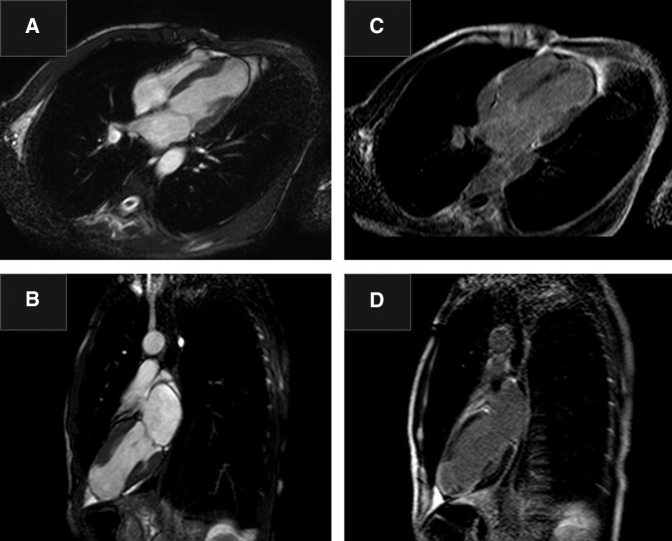
A, and B, Cardiac magnetic resonance imaging. C, and D, Delayed enhanced images.

A 52-year-old man was diagnosed with a left ventricular apical aneurysm following abnormal findings on chest X-ray. He did not complain of chest pain or shortness of breath on exertion. Twenty years ago, chest X-ray led to suspicion of sarcoidosis, but no further investigation was done, and no medication was administered. He did not have a history of chest trauma. His medical history included ulcerative colitis and type I diabetes. Electrocardiography showed a normal sinus rhythm with premature ventricular contraction, and coronary angiography revealed no significant stenosis. A computed tomography (CT) scan showed a left ventricular apical aneurysm (Figure 1, Video 1) without mural thrombus. Delayed-enhancement magnetic resonance imaging demonstrated a thin, transmurally enhanced aneurysmal wall (Figure 2). Serum blood tests did not indicate any inflammatory disease, such as sarcoidosis.

**Figure 3:**
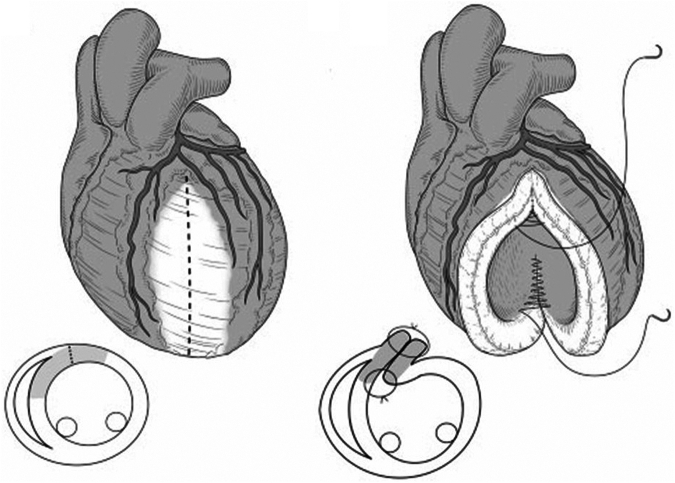
Schema of the endocardial linear infarct exclusion technique (ELIET) procedure.

The patient underwent ventricular repair with our endocardial linear infarct exclusion technique (ELIET)^1^ and cryoablation of the ventricular wall (Figure 3). After transmural linear ventriculotomy at the center of the aneurysm, cryoablation was performed between the aneurysmal wall and intact myocardium (−60°C for 2 minutes). Then the inner layer was sutured longitudinally with continuous sutures to the border between the aneurysmal wall and intact myocardium. The outer layer was closed using double layers.

**Figure 4:**
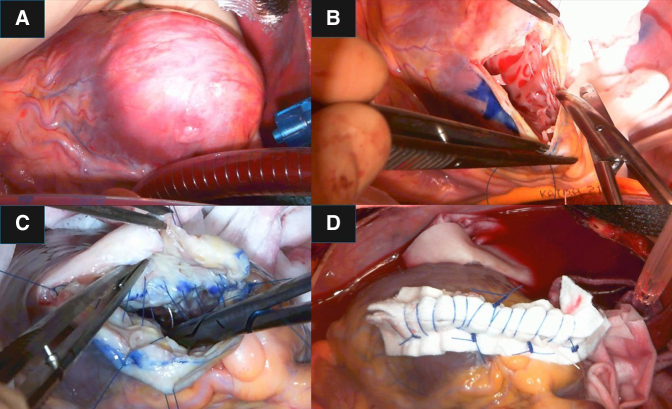
A, Intraoperative findings. B, Longitudinal incision of the aneurysm. C, Linear running suture of the inner layer between the infarcted wall and intact myocardium. D, Closure of the outer layer.

Intraoperatively, the aneurysmal wall was seen to be very thin, almost like tanned leather (Figure 4, Video 2). Histopathology showed that the aneurysm wall contained only fibrous tissue without myocardium and with no multinucleated giant cells.

**Figure 5:**
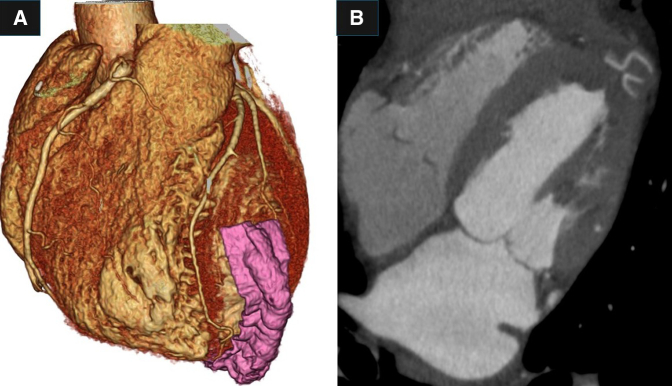
A, Three-dimensional reconstruction of postoperative computed tomography scan. B, Thin-slice image of postoperative computed tomography scan.

The patient's postoperative course was uneventful. A CT scan showed a repaired ventricular wall. The left ventricular systolic volume index was decreased from 76.2 mL/m^2^ to 49.7 mL/m^2^, and the ejection fraction was improved from 36% to 41% (Figure 5).

## Discussion

The cause of this patient's ventricular aneurysm remains unknown. In this case, there may have been a period of active inflammatory disease, such as sarcoidosis, in the past. It is known that sarcoidosis can be associated with cardiac lesions, which may resemble dilated cardiomyopathy and in some cases form left ventricular aneurysms.^2^ Lesions of cardiac sarcoidosis often occur in a patchy manner, and many of the nodules also appear in various parts of the left ventricle. The surgical indication for left ventricular aneurysm remains under debate.^3^ Treating the left ventricular aneurysm can improve LVEF, which may benefit for long-term survival. If the surgical risk is not high and the surgical procedure is expected to be reliable, we argue that surgical treatment can be considered even if the patient is asymptomatic. Additionally, with the coexistence of ulcerative colitis, it is preferable to avoid administering warfarin.

The surgical repair of ventricular aneurysms associated with sarcoidosis has been reported previously.^4^^,^^5^ Most approaches involve the use of a circular patch. In the present case, we used repaired using our method of left ventricular restoration, ELIET.^1^ The advantages are low bleeding risk, reproducibility, and coronary artery preservation. This technique can be applied not only to the anterior wall, but also to the lateral or posterior wall. As demonstrated in this case, the shape of the left ventricle improved, the risk of rupture was avoided, and the risk of left ventricular thrombus formation also decreased. Additionally, postoperative ventricular arrhythmias were no longer observed. The ELIET procedure is a useful option for left ventricular apical aneurysm without coronary artery disease.

## Conclusions

The ELIET procedure has proven effective for left ventricular aneurysms not associated with coronary artery disease.

## Conflict of Interest Statement

The authors reported no conflicts of interest.

The *Journal* policy requires editors and reviewers to disclose conflicts of interest and to decline handling or reviewing manuscripts for which they may have a conflict of interest. The editors and reviewers of this article have no conflicts of interest.

## Supplementary Data

Video 1 Four-dimensional image of computed tomography scan. Video available at: https://www.jtcvs.org/article/S2666-2507(25)00268-8/fulltext

Video 2 The endocardial linear infarct exclusion technique (ELIET) procedure. Video available at: https://www.jtcvs.org/article/S2666-2507(25)00268-8/fulltext.
