# Supplementary figures and images for: Surgical repair for left ventricular apical aneurysm without coronary artery disease
Source: JTCVS Tech. 2025 Jun 28;33:142–5. doi: 10.1016/j.xjtc.2025.06.019 (PMC12529692; doi:10.1016/j.xjtc.2025.06.019)

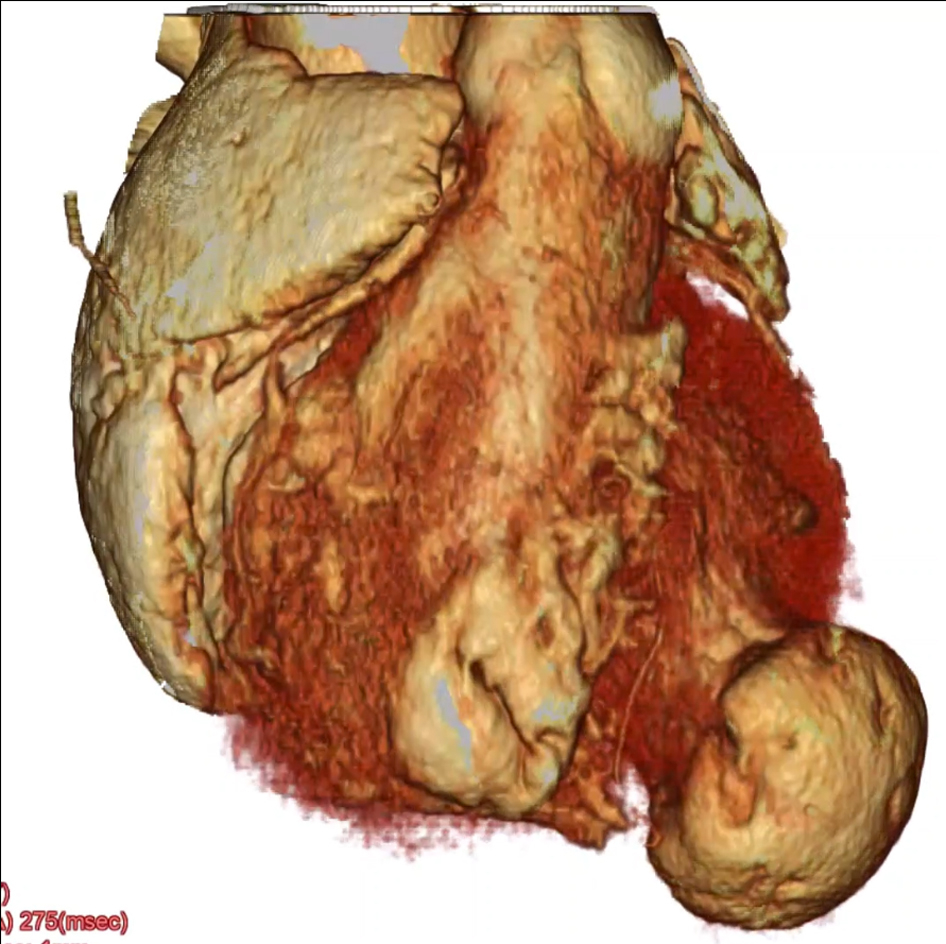

Supplement: Video 1 — Four-dimensional image of computed tomography scan. Video available at: https://www.jtcvs.org/article/S2666-2507(25)00268-8/fulltext [file fx2.jpg]

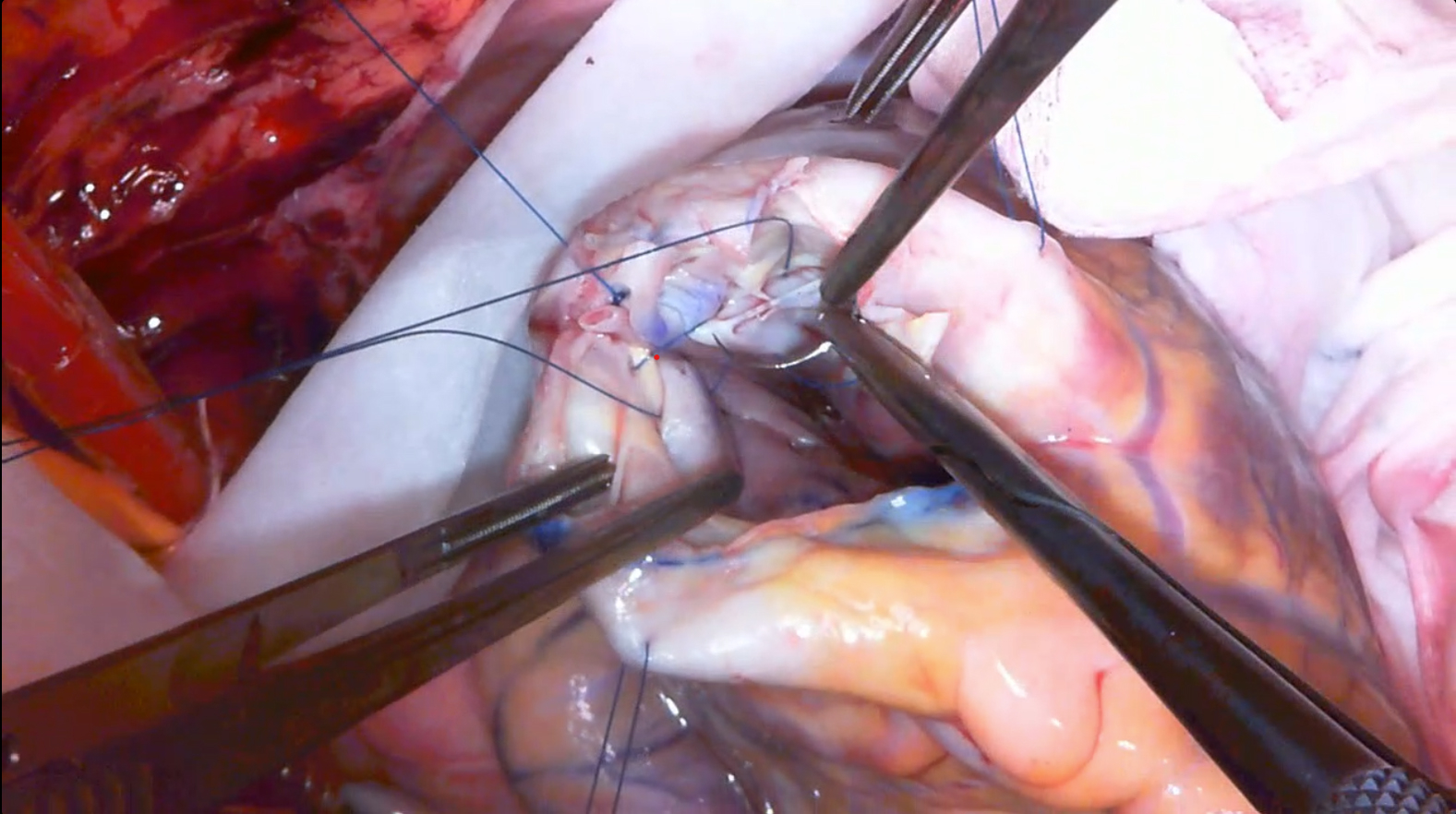

Supplement: Video 2 — The endocardial linear infarct exclusion technique (ELIET) procedure. Video available at: https://www.jtcvs.org/article/S2666-2507(25)00268-8/fulltext. [file fx3.jpg]
